# IgG4-Related Disease in Childhood: Clinical Presentation, Management, and Diagnostic Challenges

**DOI:** 10.3390/children12070888

**Published:** 2025-07-05

**Authors:** Silvia Taranto, Luca Bernardo, Angela Mauro, Anna Perrone, Agnese Tamborino, Teresa Giani

**Affiliations:** 1 Pediatric Department, “Vittore Buzzi” Children’s Hospital, 20154 Milan, Italy; silvia.taranto@unimi.it; 2 Pediatric Unit, Department of Childhood and Developmental Medicine, Fatebenefratelli-Sacco Hospital, 20121 Milan, Italy; luca.bernardo@asst-fbf-sacco.it; 3 Pediatric Rheumatology Unit, Department of Childhood and Developmental Medicine, Fatebenefratelli-Sacco Hospital, 20121 Milan, Italy; 4 Radiology Unit, Meyer Children’s Hospital IRCCS, 50139 Florence, Italy; anna.perrone@meyer.it; 5 Infectious Disease Unit, Meyer Children’s Hospital IRCCS, 50139 Florence, Italy; agnese.tamborino@meyer.it; 6 Department of Pediatrics, Meyer Children’s Hospital IRCCS, 50139 Florence, Italy; teresa.giani@meyer.it

**Keywords:** immunoglobulin G4-related disease, autoimmune related systemic disease, orbital disease, child

## Abstract

Immunoglobulin G4-related disease (IgG4-RD) is an immune-mediated fibroinflammatory disorder primarily affecting adults. The disease in pediatric age is unusual and preferentially affects adolescents. In contrast to adults, who commonly exhibit the involvement of multiple organs simultaneously or sequentially over time, young patients tend to present with a localized disease, typically affecting the orbits. Proptosis, ptosis, diplopia, and restricted eye movement may be observed in these patients. Symptoms are proteiform, and the disease is chronic and indolent with a relapsing–remitting course. Diagnostic criteria have been developed for adults, which may not fully capture the pediatric disease phenotype. If untreated or poorly managed, IgG4-RD can lead to progressive fibrosis and scarring of affected organs, potentially causing irreversible damage. We conducted a narrative review using the IMRAD approach, presenting a nonsystematic analysis of the literature on pediatric IgG4-RD. Original papers, case reports/series, and relevant reviews in English were selected from PubMed, EMBASE, and Web of Science up to January 2024. Keywords included “IgG4-Related Disease” and “pediatric” and, additionally, we presented two original pediatric cases. Our purpose is to offer an overview of IgG4-RD manifestations, and challenges in diagnosing and managing this rare condition in children.

## 1. Introduction

Immunoglobulin G4-related disease (IgG4-RD) is a multi-organ, immune-mediated fibroinflammatory disease of unknown etiology. The typical histological findings are lymphoplasmacytic infiltration, mostly characterized by IgG4-positive plasma cells, and a fibrosis with a typical “storiform” pattern [1,2].

This is a relatively recently recognized condition, first described in the early 2000s. In recent years, there has been a progressive increase in the number of diagnoses due to improved knowledge [3,4]. In the USA, during the period 2015–2019, the incidence has raised from 0.78 to 1.39 per 100,000 person-years, with a prevalence of 5.3/100,000 persons [5,6]. A slight female predominance (57.6%) has been observed in the USA, in contrast with previous Japanese data [5]. The average age at diagnosis worldwide is 50–70 years, while pediatric cases have been rarely reported [7].

The disease presentation is protean, with an indolent and slowly progressive clinical course, affecting either a single organ or body area or manifesting as a multisystemic condition. The disease progression results in substantial tissue damage, accompanied by organomegaly and functional failure [3]. Pancreas, kidney, orbits, lacrimal, and salivary glands, lungs, pleura, and peritoneum are among the most commonly affected organs and tissues [1].

An increase in serum IgG4 levels is a common, although unspecific, sign. IgG4 levels usually correlate with the disease severity and with the affected anatomic region [8]. Elevated IgG and IgE, peripheral eosinophilia, raised Erythrocyte Sedimentation Rate (ESR) and C-reactive protein (CRP) level, positive antinuclear antibodies (ANAs) and Rheumatoid Factor (RF), and hypocomplementemia are additional common findings [9]. However, these laboratory alterations are not pathognomonic and may overlap with other immune-mediated or infectious diseases. The presence of lymphoplasmacytic infiltration, storiform fibrosis, and obliterative phlebitis is highly suggestive of the diagnosis, with an increased number of IgG4+ plasma cells in affected tissues. However, obtaining biopsy samples can be challenging, particularly in pediatric patients, where invasive procedures may not always be feasible. In such cases, a combination of clinical, serological, and radiological findings is used to support the diagnosis, provided that other conditions are excluded.

The first set of diagnostic criteria, the Comprehensive Diagnostic Criteria (CDC), was defined in 2011 by the Japanese IgG4 team. However, as IgG4-RD gained widespread acceptance in the following years, several problems arose in clinical practice, including difficulty in obtaining biopsy samples and defining the serum IgG4 cutoff level, as well as the impaired immunostaining of IgG4. In light of these challenges, the Japanese IgG4 team updated the 2011 CDC, proposing the 2020 revised comprehensive diagnostic (RCD) criteria for IgG4-RD. These criteria consist of three domains: (1) clinical and radiological features, (2) serological diagnosis, and (3) pathological diagnosis (Table 1) [10].

Concurrently, in 2019, the American College of Rheumatology (ACR) and the European League Against Rheumatism (EULAR) have developed classification criteria for research purposes (Table 2) [11]. While these criteria are useful in identifying patients for clinical studies, their applicability in routine clinical practice, particularly in children, remains uncertain. In fact, well-established and standardized criteria for pediatric patients are still lacking, and current diagnosis relies on a combination of clinical, laboratory, and imaging findings, often referencing criteria developed for adults [7]. Given these limitations, the exclusion of other diseases with overlapping features, such as infectious, neoplastic, and other immune-mediated disorders, is a crucial step before confirming the diagnosis [12]. Radiological findings, particularly on CT and MRI, can provide supportive evidence, showing soft tissue enlargement, organ hypertrophy, and perivascular involvement. PET-CT has also been proposed as a useful tool for detecting multisystemic involvement and monitoring disease activity. However, imaging findings alone are insufficient for diagnosis and must always be correlated with clinical and histological data.

Due to these challenges, a multidisciplinary approach involving rheumatologists, immunologists, radiologists, and pathologists is often necessary to achieve an accurate diagnosis, especially in pediatric cases where the disease is rare and may present atypically.

Early diagnosis and subsequently early treatment are crucial to preventing fibrotic changes and their implications [13]. We conducted a narrative review of the current literature on this condition in children, discussing the challenges in diagnosing and managing IgG4-RD in this population. Additionally, we describe two diagnostically challenging cases. Both required a meticulous differential diagnosis and prolonged follow-up to reach a possible diagnosis of IgG4-RD. In one case, histopathology was inconclusive, likely due to the analysis of bone tissue, while in the other, the family declined biopsy. These cases illustrate the complexity of diagnosing IgG4-related disease in children and highlight the need for a thorough, multidisciplinary approach when histological confirmation is not feasible.

## 2. Materials and Methods 

This narrative review was performed in accordance with the IMRAD (Introduction, Methods, Results, and Discussion) approach and presented a nonsystematic analysis of the literature on the pediatric IgG4-related disease [14]. The authors considered original scientific papers, case reports/series, and reviews of major relevance in the English language published until 20 January 2024. PubMed, EMBASE, and Web of Science were utilized as electronic databases for this research.

The following keywords (alone and/or in combination) were used to select relevant articles: “IgG4-Associated Autoimmune Disease”, “IgG4-Related Disease”, “Autoimmune related systemic disease”, “IgG4 Related Disease”, “IgG4-RD” AND “pediatric” or “children” or “child” or “adolescent”.

All cases included in this review were originally classified as IgG4-RD by the authors of the respective publications. To ensure consistency and transparency, we evaluated these cases based on fundamental criteria common to the three main classification systems for IgG4-RD: the 2019 ACR/EULAR classification criteria [11], the 2011 Comprehensive Diagnostic Criteria (CDC), and the 2011 Comprehensive Clinical Diagnostic Criteria (CCDC) [15]. Specifically, we assessed the exclusion of alternative diagnoses, the presence of organ involvement with characteristic clinical and radiological manifestations, serological findings, and histopathological confirmation. These key aspects have been summarized in Table 1 for clarity. The contributions were independently reviewed by two researchers. In cases of uncertainty, a third reviewer was consulted for additional assessment. The resulting draft was then critically revised and approved by all.

Case presentation 1

**Figure 1:**
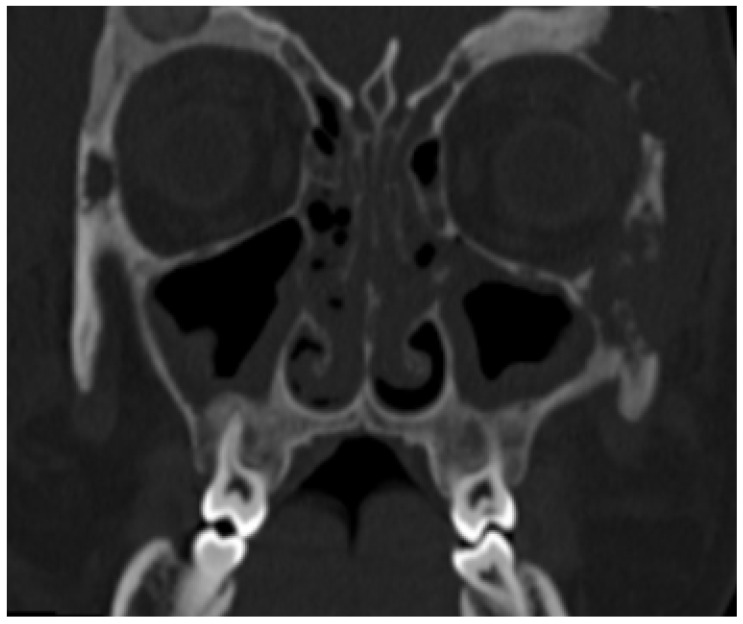
Multiple, partially confluent, osteolithic lesions in zygomatic and frontal bones. Paranasal sinus involvement.

A 12-year-old Caucasian girl was admitted to the emergency department for a prolonged and fluctuating asymptomatic swelling and redness of her left upper eyelid, which started about six months earlier. The girl was initially treated with local antibiotics, and then, after about two months, she was hospitalized. A computed tomography (CT) scan detected signs of palpebral cellulitis, maxillary sinusitis, and lytic lesions on the lateral side of the orbit and the left frontal bone (Figure 1).

While routine laboratory exams were inconclusive, immunological investigations revealed a reduction in Natural Killer (NK) cells (89/µL, 5%) and elevated serum IgG (1597 mg/dL) and IgG4 (223 mg/dL). Flow cytometry also showed normal levels of CD8+ T cells and no abnormal expression of PD-1. Despite the absence of fever and other systemic and laboratory signs of inflammation, suspecting a bacterial infection, broad-spectrum intravenous antibiotics were prescribed for 2 weeks, followed by an oral antibiotic treatment for an additional 3 weeks.

**Figure 2:**
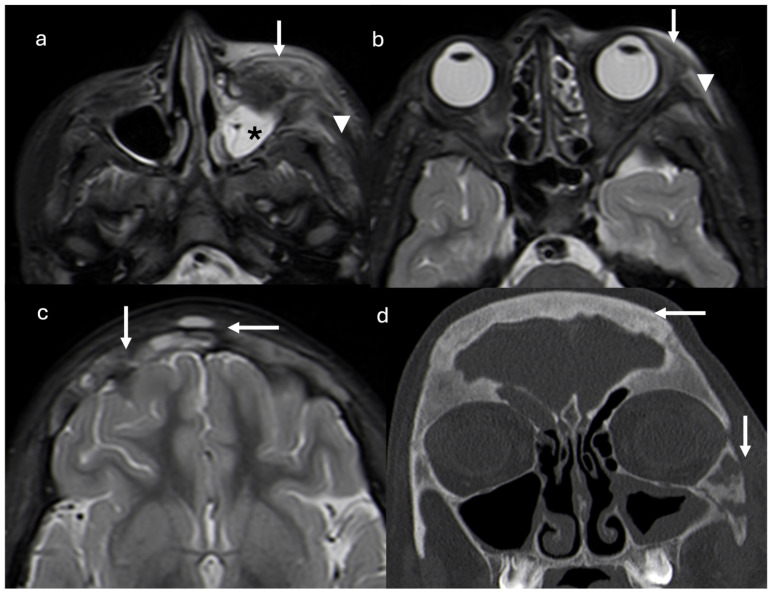
Axial short tau inversion recovery (STIR) magnetic resonance images show diffuse mucosal thickening of left maxillary sinus (asterisk), periorbital adipose tissue inflammation and pre-septal cellulitis (arrows), and temporal and masseter muscle edema (arrowheads) (**a**,**b**). Thickening and diffuse signal alterations of frontal bone with epicranic soft tissue involvement (arrows) are demonstrated (**c**). Coronal reconstruction CT scan (**d**) confirmed thickening on frontal and zygomatic bones with lytic areas. Right frontal and left maxillary sinus inflammation is present (arrows).

The follow-up CT scan showed lytic lesions at the frontal and zygomatic bones, and a magnetic resonance imaging (MRI) scan was required. One month after discontinuation of antibiotic therapy, MRI revealed a diffuse mucosal thickening of the left maxillary sinus, periorbital adipose tissue inflammation, pre-septal cellulitis, temporal and masseter muscle edema, and thickening and diffuse signal alterations of frontal bone with epicranic soft tissue involvement (Figure 2).

The family sought a second opinion at our hospital.

At a physical exam, her upper left eyelid appeared hyperemic and moderately edematous, not painful or tender to palpation, with unrestricted ocular movements. 

Three months later, a second MRI showed a complete muco-inflammatory pansinusitis, bilateral orbital and left zygomatic bone erosions, mild left dacryoadenitis, left masseter muscle oedema, and left pre-septal orbital fat inflammation. On the same side, inflammation was also appreciable in the periorbital adipose tissue, orbicularis, temporal, and masseter muscles, with additional lytic bone lesions present on the zygomatic and frontal bones, and fibroinflammatory dural thickening.

Routine laboratory examination results were negative or within normal ranges. Elevated levels of total IgG and IgG4 in the serum were confirmed (2360 mg/dL, normal values nv < 1909, and 353 mg/dL, nv < 135 respectively).

A functional endoscopic sinus surgery (FESS) was performed in order to treat her chronic sinusitis and biopsy the maxillary sinus mucosae and the left zygomatic bone. Mild fibroblastic and myofibroblastic mucosal proliferation were detected, along with a moderate trabecular reorganization of bone tissue and a mild fibrosclerosis of the bone marrow. The biopsy showed an osteoblastic rim around isolated bone spicules and mild perivascular lympho-monocytic infiltrate. Hemorrhagic extravasations with hemosiderin pigment were also present. Immunostains performed included CD38, IgG4, CD163, p16, CD34, and LCA, with two IgG4-positive cells and a low IgG4/IgG ratio reported. As unequivocal histological support was lacking in this patient, a careful differential diagnosis was conducted, with multiple consultations from infectious disease specialists, immunologists, and oncohematologists during her various hospital admissions. Although the presence of lytic bone lesions initially raised the suspicion of histiocytosis, a series of assessments ultimately led us to favor IgG4-RD. Factors suggesting this diagnosis included the indolent and prolonged course of the disease, characterized by intermittent episodes of mild hyperemia and swelling of the periorbital soft tissues and ipsilateral hemiface, without pain or other symptoms, and the absence of systemic and laboratory signs of inflammation. Additionally, radiological studies revealed the typical head and neck distribution of IgG4-RD observed in adolescent patients, involving areas such as the sinus mucosa, lacrimal gland, adipose tissue, and dural membrane. Bone lesions appeared adjacent to each other, partially confluent, and located near the altered paranasal sinuses. In histiocytosis, bone lesions are well-defined and less destructive and are often multiple, with a less extensive involvement of adjacent soft tissues. Elevated serum IgG and IgG4 levels, the absence of granulomas and histiocytic proliferation in the biopsy, and lack of involvement of other organs further supported IgG4-RD over histiocytosis.

Corticosteroid oral treatment was started, leading to a rapid clinical and radiological improvement. Nevertheless, the patient chose to discontinue the follow-up after three months.

Case presentation 2

**Figure 3:**
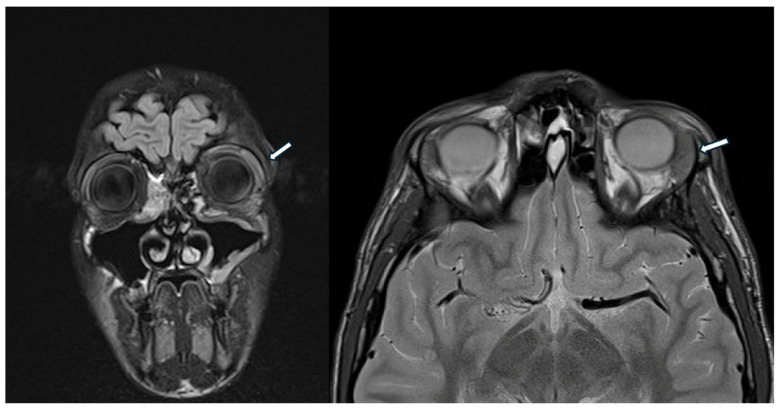
The head MRI shows enlarged left lacrimal gland (white arrow) and minimal peribulbar fluid.

A previously healthy 14-year-old male was referred to our pediatric rheumatology outpatient clinic for recurrent dacryoadenitis. This condition had been diagnosed three years earlier at a local ophthalmological center, where the patient presented with bulbar conjunctival hyperemia and eyeball pain and underwent bulbar ultrasound, optical coherence tomography (OCT), and cranial CT scan, revealing peripalpebral soft tissue swelling on the left portion of the eye. A beneficial treatment with oral and topical antibiotic therapy, along with topical corticosteroids, was initially prescribed. Additionally, corrective lenses have been prescribed for simple hypermetropic astigmatism. However, due to the recurrence of inflammatory episodes coinciding with the tapering of corticosteroids, an orbital MRI was performed, revealing an enlarged left lacrimal gland and minimal peribulbar fluid (Figure 3).

Routine blood exams provided uninformative results, except for increased IgG4 levels (2970 mg/L, nv < 1350 mg/L). Suspecting systemic IgG4-RD, the boy was referred to our pediatric rheumatology clinic.

At the physical examination, the patient was in good general condition, displaying swelling and mild hyperemia of the left eye. Comprehensive laboratory analyses confirmed elevated IgG4 levels (1598 mg/L). Abdominal and neck ultrasound was negative. Due to persistent ocular symptoms and signs such as prominence of the left lacrimal gland with lacrimation and conjunctival hyperemia, the ophthalmologist recommended a left lacrimal gland biopsy, which the parents refused. At the follow-up examinations, IgG4 levels were persistently elevated (2282 mg/L), with negative inflammatory indices and blood counts within a normal range.

The principal features of the two cases are reported in Table 3.

## 3. Results

We collected a total of one-hundred and seventeen cases of pediatric IgG4-RD: fifty-nine cases were described in a review published in 2023 by Saad et al. [16], fifty-six cases were reported in the literature separately, and finally, two new cases were diagnosed at our facilities. Of these, twelve cases were excluded due to insufficient diagnostic confirmation: ten lacked a biopsy [17,18,19,20,21,22,23,24], one had a negative histological result [25] and one had borderline findings [26]. Thus, 105 cases were included in the final analysis. Consistently with previous data, the mean age at presentation was 11 years (range 1–18 years old), with a slight predominance of males (53%), contrasting with the findings of Bu et al., who reported a female predominance [27]. Regarding the clinical presentation, 71 patients (68%) had localized disease (with a single organ involvement), and 34 (32%) had a systemic disease. Among the one-hundred and five patients included in this study, orbital disease was present in forty-two of one-hundred and five cases (40%), with thirty cases (28%) limited to the orbits (IgG4-related orbital disease or IgG4-ROD); eight patients (7%) with hepatic involvement, nine (8%) with pancreatic manifestations, three (3%) with pulmonary disease, and nine (7%) with renal involvement. 

A total of 52% of cases presented with constitutional symptoms and 52% had elevated inflammatory markers. In 80% of patients, IgG4 levels were increased, fulfilling the serological criteria. A total of 100% of cases had histological findings consistent with IgG4-related disease due to the inclusion criteria. Overall, 80% met both serological and histological criteria, supporting a definitive diagnosis. Steroid treatment was employed in 81% of patients.

The main characteristics of the one-hundred and three pediatric IgG4-RD cases described in the literature, and the two children we described are reported in Table 4.

## 4. Discussion

IgG4-RD is a rare and often underestimated condition in pediatric patients, primarily affecting adolescents [3,7,102]. The clinical presentation is typically subtle, with symptoms beginning months or even years before diagnosis [102,103].

In adults, constitutional symptoms are rare. Weight loss, if present, is usually mild, with the exception of IgG4-related autoimmune pancreatitis. Fever is a highly atypical symptom [103]. ESR can be elevated, due to high serum immunoglobulin levels, while increased CRP is less common [3]. Low serum levels of ANA and positive RF are frequent, although serum positivity for more specific autoantibodies is uncommon [103].

In children, systemic constitutional symptoms are more frequently observed, and half of the cases may show an increase in inflammatory marker [7,17].

According to the existing literature, approximately 40% of the adult patients have single organ involvement, with the disease commonly affecting the pancreas, lymph nodes, orbit, and salivary tract [9,103]. Organ-specific symptoms at diagnosis frequently include abdominal pain, sicca syndrome features, respiratory symptoms, pruritus, and diarrhea [103].

Central nervous system (CNS) involvement is rare and is typically localized to the pituitary gland and meninges, sparing the parenchyma [104,105].

Single organ involvement is prevalent in children, occurring in approximately 60% of cases according to the systematic review by Karim et al. In our analysis, 68% of the 105 cases presented as localized disease [7].

The pancreato-hepatobiliary tract, lymph nodes, salivary glands, and lungs can be affected in children [21]. However, the orbits are the most frequently involved area, with a prevalence of 38% among the 59 cases described by Bu et al., and 40% among the 105 cases we analyzed [27]. According to Bu et al., pediatric IgG4-ROD rarely presents bilaterally (15%), and extra-orbital involvement is infrequent (20%), contrasting with adults, where it occurs in 70% of cases [27].

In a study of 13 children, 85% exhibited unilateral orbital protrusion/swelling, with 46% showing eyelid involvement, whereas dacryoadenitis represents the most prevalent manifestation in adults [30].

Both possible IgG4-RD adolescents described presented with localized disease. The boy had orbital involvement, while the female patient exhibited a more extensive condition affecting the lacrimal gland, maxillary and ethmoidal sinuses, periorbital adipose tissue, orbicularis, temporal, and masseter muscles, zygomatic and frontal bones, and leptomeninges. Hypertrophic pachymeningitis (IgG4-HP) is an uncommon manifestation of IgG4-RD, exceptionally reported in pediatric cases, with only two patients described so far [36,104,106,107,108]. Clinically, it may remain asymptomatic, as seen in the girl we described, or manifest with chronic headache, sometimes accompanied by a wide range of neurological symptoms resulting from mass effect, nerve compression, or vascular compression [109]. It usually presents in isolation, without other organ involvement, normal-to-low serum IgG4 levels, and no systemic symptoms, posing differential diagnosis challenges [110]. Brain MRI may reveal contrast enhancement and localized or diffused thickening of the dura mater [104,111]. The cerebrospinal fluid (CSF) usually shows only mild and unspecific alterations [112]. Increased CSF IgG4 levels have been only occasionally reported and can be diagnostically valuable when combined with suggestive histological features [30]. In the case of the girl we described, this neurological manifestation was detected on the MRI of the head, although a CSF analysis or biopsy examination was not performed.

This young girl also exhibited bone erosion in the zygomatic and frontal bones. This is another uncommon feature of IgG4-RD, rarely reported in the literature and primarily involving the skull bones and spine [113,114,115].

In the same 12-year-old patient, widespread sinusitis was documented on imaging but was completely asymptomatic. Although the typical classification or diagnostic criteria do not usually include the sinonasal regions, their involvement is reported in a percentage ranging from 32% to 65% of the cases [116,117,118]. Typically, sinusitis manifests with evocative features such as nasal congestion, facial pain or pressure, and a long-lasting diminished sense of smell. Nasal polyps may be observed on endoscopy, and neuroimaging may reveal bilateral and diffuse involvement of the paranasal sinuses with sinus wall thickening [116].

Our two patients lacked constitutional symptoms and inflammatory markers but exhibited elevated serum IgG4 levels.

Around 20% of adults classified as IgG4-RD according to the 2019 ACR/EULAR IgG4-RD classification criteria show normal serum IgG4 levels [11]. Furthermore, 9% of patients do not undergo biopsy, and in 37% of those who do undergo biopsy, the classic histopathologic findings are missing [11].

Due to the lack of specific pediatric criteria, diagnosis relies on criteria established for adults, particularly the RCD Criteria [10]. However, typical IgG4-RD features such as IgG4-positive plasma cell counts, focally dense plasma cell infiltrate at biopsy, and specific IgG4 ratio are not universally observed in children as well [27]. Normal serum IgG4 levels are reported in a significant percentage of children, mostly in those with orbital involvement (58% in pediatric IgG4-ROD vs. 33% in pediatric IgG4-RD as a whole) [27]. Additionally, typical histological features, especially storiform fibrosis and obliterative phlebitis, are observed only in a minority of young patients [17,27].

In the girl described, who underwent a biopsy, characteristic histological findings were absent, except for the presence of a modest fibrous component.

Therefore, the possible diagnosis in both reported cases was established following the careful exclusion of other similar conditions and was primarily supported by the prolonged and indolent disease course, elevated serum IgG4 levels, and orbital localization.

Early treatment is crucial to prevent organ damage, especially considering that children may have a longer disease duration and the consequences of fibrosis can be more impactful [13]. Therapy involves immunosuppressive agents for both remission induction and, given the relapsing nature of the disease, for remission maintenance as well [119]. Not all manifestations of the disease require immediate treatment, because some indolent cases can persist for decades; therefore, watchful waiting may be an option in specific situations [102].

Glucocorticoids (GCs) are nearly universally used as first-line therapy [1,102,119]. The treatment usually involves prednisone at a dosage of 0.5–2 mg/kg/day for 2–4 weeks, followed by a gradual tapering. [7,119]. Clinical improvement typically occurs rapidly, although the speed can vary depending on the affected organs and the extent of fibrosis [101]. In cases where the diagnosis is uncertain, swift improvement following GCs is often considered a valuable diagnostic indicator [1]. Recurrence rates after treatment with steroids are significant: in adults, they range from 10% to 53%, while in the pediatric population, they reach up 57% of cases [1,7]. In cases of relapse or GC resistance, second-line treatments such as mycophenolate mofetil, azathioprine, methotrexate, rituximab, or surgery should be considered [120]. Rituximab has recently been recognized as an effective treatment option, both for remission induction and for treatment of disease flares, and, together with GC, it represents the most established treatment option [121]. Immunosuppressants are usually introduced early, reducing the likelihood of relapse and the development of GC toxicity [122].

Beyond rituximab, alternative biologics such as adalimumab or ruxolitinib have been explored in severe or refractory cases, though evidence remains limited [123].

Combination therapy, particularly glucocorticoids with steroid-sparing agents such as azathioprine or mycophenolate mofetil, has shown improved disease control and reduced relapse rates in pediatric cases [123]. Our first case successfully responded to first-line treatment, although long-term outcome details are not available, while the girl developed a recurrence of inflammation during the tapering of GC therapy.

In both cases, histopathology was not conclusive in confirming IgG4-RD. In the adolescent girl, the biopsy did not provide definitive support for the diagnosis, possibly due to the nature of the sampled tissue, which was predominantly bone. In the boy, a histopathological sample was not obtained due to parental refusal. Nevertheless, in both patients, the diagnosis of possible IgG4-RD was supported by a prolonged and indolent clinical course, characteristic organ involvement, persistently elevated IgG4 levels, exclusion of alternative diagnoses, and a positive response to corticosteroid therapy. These elements align with recognized diagnostic frameworks for IgG4-RD. Given the challenges in obtaining biopsies in pediatric patients, particularly in delicate anatomical sites, a multidisciplinary approach integrating clinical, serological, and radiological findings is often necessary for diagnosis. The description of these two cases, despite the lack of definitive histopathological confirmation, illustrates the real-world difficulties in clinical practice. These cases underscore the importance of an integrated diagnostic strategy in pediatric IgG4-RD, where biopsy may not always be feasible.

## 5. Conclusions

This work offered an overview of IgG4-related disease in pediatric age, a condition that is extremely rare in this age group. IgG4-RD in children often shows a localized distribution, preferentially affecting the orbits. The disease course is chronic and indolent, occasionally accompanied by constitutional symptoms. Elevated IgG4 serum levels are an evocative laboratory sign of this condition. Although typical histological findings provide a definitive diagnosis, they may be sometimes unclear or absent. Additionally, performing biopsies in children can be challenging due to the invasiveness of the procedure and the need for sedation. These challenges underline the need for further studies to better shape diagnostic criteria in this population. GCs are the cornerstone of IgG4-RD therapy, especially in the initial stages. However, given the chronic nature of this condition, characterized by a relapsing–remitting course, the early introduction of immunosuppressive agents is often required to manage long-term treatment and minimize potential GC side effects.

## Figures and Tables

**Table 1:** The 2020 revised comprehensive diagnostic (RCD) criteria for IgG4-RD [10].

[Item 1] clinical and radiological features
One or more organs show diffuse or localized swelling or a mass or nodule characteristic of IgG4-RD. In single organ involvement, lymph node swelling is omitted.
[Item 2] serological diagnosis
Serum IgG4 levels greater than 135 mg/dL.
[Item 3] pathological diagnosis
Positivity for two of the following three criteria:
- Dense lymphocyte and plasma cell infiltration with fibrosis.
- Ratio of IgG4-positive plasma cells/IgG-positive cells greater than 40% and the number of IgG4-positive plasma cells greater than 10 per high powered field.
- Typical tissue fibrosis, particularly storiform fibrosis, or obliterative phlebitis.
Diagnosis: definite: (1) + (2) + (3) probable: (1) + (3): possible: (1) + (2).

**Table 2:** Summary of the diagnostic criteria for IgG4-related disease, adapted from Wallace et al. 2019 ACR/EULAR classification [11].

Yes or No	1. Entry criteria
	Clinical or radiologic signs typical of involvement in a characteristic organ (such as the pancreas, salivary glands, bile ducts, orbits, kidneys, lungs, aorta, retroperitoneum, pachymeninges, or thyroid gland [e.g., Riedel’s thyroiditis]) OR histopathologic evidence of inflammation with a lymphoplasmacytic infiltrate of unknown cause in any of these organs.
Yes or No	2. Exclusion criteria: domains and items
	Clinical
	Fever
	Absence of an objective therapeutic response to glucocorticoids
	Serologic
	Unexplained leukopenia and thrombocytopenia
	Peripheral eosinophilia
	Positive antineutrophil cytoplasmic antibodies (ANCAs), specifically targeting proteinase 3 or myeloperoxidase
	Presence of SSA/Ro or SSB/La antibodies
	Positive for double-stranded DNA, RNP, or Sm antibodies
	Detection of other disease-specific autoantibodies
	Presence of cryoglobulins (cryoglobulinemia)
	Radiologic
	Radiologic findings suggestive of malignancy or infection that remain inadequately evaluated
	Rapid progression observed on imaging studies
	Long bone lesions characteristic of Erdheim–Chester disease
	Enlarged spleen (splenomegaly)
	Pathologic
	Cellular infiltrates suspicious for malignancy that have not been thoroughly investigated
	Immunohistochemical or molecular markers indicative of inflammatory myofibroblastic tumor
	Marked neutrophilic inflammatory response
	Evidence of necrotizing vasculitis
	Extensive or prominent tissue necrosis
	Predominantly granulomatous inflammation
	Histopathologic features consistent with a macrophage or histiocytic disorder
	Established diagnosis of one of the following conditions:
	Multicentric Castleman’s disease
	Crohn’s disease or ulcerative colitis (when limited to pancreatobiliary involvement)
	Hashimoto’s thyroiditis (when confined to the thyroid gland)
	If case meets entry criteria and does not meet any exclusion criteria, proceed to point 3.
	3. Inclusion criteria: domains and items
	Histopathology
0	Uninformative biopsy
+4	Dense lymphocytic infiltrate
+6	Dense lymphocytic infiltrate and obliterative phlebitis
+13	Dense lymphocytic infiltrate and storiform fibrosis with or without obliterative phlebitis
0–16, as follows:	Immunostaining
Assigned weight is 0 if the IgG4+:IgG+ ratio is 0–40% or indeterminate and the number of IgG4+ cells/hpf is 0–9.g Assigned weight is 7 if (1) the IgG4+:IgG+ ratio is ≥41% and the number of IgG4+ cells/hpf is 0–9 or indeterminate; or (2) the IgG4+:IgG+ ratio is 0–40% or indeterminate and the number of IgG4+ cells/hpf is ≥10 or indeterminate.	
Assigned weight is 14 if (1) the IgG4+:IgG+ ratio is 41–70% and the number of IgG4+ cells/hpf is ≥10; or (2) the IgG4+:IgG+ ratio is ≥71% and the number of IgG4+ cells/hpf is 10–50.	
Assigned weight is 16 if the IgG4+:IgG+ ratio is ≥71% and the number of IgG4+ cells/hpf is ≥51.	
	Serum IgG4 concentration
0	Normal or not checked
+4	>Normal but <2× upper limit of normal
+6	2–5× upper limit of normal
+11	>5× upper limit of normal
	Involvement of lacrimal, parotid, sublingual, and submandibular glands (bilaterally):
0	No glandular involvement
+6	Involvement of one glandular group
+14	Involvement of two or more glandular groups
	Chest
0	Not assessed or neither finding is present
+4	Peribronchovascular and interlobular septal thickening
+10	Paravertebral, band-like soft tissue within the thoracic cavity
	Pancreas and Biliary Tree
0	Not assessed or no listed abnormalities present
+8	Diffuse pancreatic enlargement with loss of normal lobulated contour
+11	Diffuse pancreatic enlargement with a capsule-like rim showing decreased enhancement
+19	Involvement of both the pancreas (as described above) and the biliary tree
	Kidney
0	Not assessed or no listed findings present
+6	Hypocomplementemia
+8	Thickening or soft tissue involving the renal pelvis
+10	Bilateral low-attenuation areas in the renal cortex
	Retroperitoneum
0	Not assessed or neither finding is present
+4	Diffuse thickening of the abdominal aortic wall
+8	Circumferential or anterolateral soft tissue surrounding the infrarenal aorta or iliac arteries
	4: Total inclusion points
	A case qualifies for classification as IgG4-related disease (IgG4-RD) if the entry criteria are fulfilled, no exclusion criteria apply, and the total score is 20 or higher.

**Table 3:** Principal features of the two cases we present.

Second Case	First Case	
14 yy, M	12 yy, F	Epidemiology
orbital involvement-dacryodenitis	orbital involvement-orbital swelling	Clinical presentation
elevated IgG4 levels	elevated IgG4 levels	Laboratory findings
CT and MRI: inflammation (swelling and free fluid)	CT and MRI: inflammation of tissues and lytic bone lesions	Radiological findings
not performed	absence of typical histological findings	Histological findings
oral corticosteroid	oral corticosteroid	Treatment
recurrence of disease with tapering of the corticosteroids	rapid clinical and radiological improvement	Outcome

Abbreviations: yy: years old, mm: months old, F: female, M: male, CT: computed tomography, MRI: magnetic resonance imaging.

**Table 4:** Cases of pediatric IgG4-RD reported in the literature.

Outcome	Treatment	Histological Criteria	Serum IgG4 Level	Acute Phase Reactants	Constitutional Symptoms	Clinical and Radiological Manifestations	Age-Gender	Authors
Improvement	Steroids, MTX	Present	Elevated	Normal	Yes	Orbital disease colitis	16 yy F	Tille et al., 2020 [28]
Remission	Steroids	Present	Elevated	Elevated	Yes	Tumor of the orbit and pterygopalatine fossa Lymphadenopathy	13 yy M	Dylewska et al., 2020 [29]
Remission	Steroids	Present	Elevated	Normal	No	Orbital disease	4 yy M	Smerla et al., 2018 [30]
Normalization of liver enzymes, no relapses	Steroids, AZA	Present	NR	NR	NR	Autoimmune hepatitis	10 yy ± 3-3 M /3 F	Aydemir et al., 2019 [31]
Resolution on MTX and Infliximab	Steroids, AZA, tacrolimus, MTX, infliximab	Present	Elevated	NR	Yes	Pancreatitis Hepatitis Colitis Lymphadenopathy	11 yy F	Bolia et al., 2016 [25]
Normalization of LFTs, radiographic regression	Steroids, AZA, UDCA	Present	Normal	NR	Yes	Pancreatitis AIHA/hepatitis	7 yy M	
NR	Surgical	Present	NR	NR	No	Chronic sclerosing sialadenitis (CSS) or Küttner tumor (left neck mass)	15 yy F	Keidar et al., 2020 [32]
NR	NR	Present	Elevated	NR	Yes	Cough, epistaxis, nasal swelling, nasal mass	9 yy F	Namireddy et al., 2021 [33]
Symptomatic, reduction in the size of the mass, and decrease of serum IgG4 levels	Steroids	Present	Elevated	Elevated	No	Failure to thrive, recurrent respiratory infections Mediastinal lymphadenopathies, posterior mediastinal mass	22 mm F	Corujeira et al., 2015 [34]
NR	Steroids, AZA + Nephrectomy	Present	NR	NR	Yes	Skin lesions, necrotizing vasculitis, recurrent uveitis Left kidney tumor	7 yy M	Nastri et al., 2018 [35]
NR	Surgical	Present	Normal	NR	NR	Focal seizures—large mass in the left frontoparietal region	16 yy M	Nambirajan et al., 2019 [36]
NR	NR	Present	Elevated	NR	NR	Episcleritis, palpable purpura, salivary gland enlargement, and bloody diarrhea, focal mass in the pancreatic tail, Renal necrotizing granulomatous vasculitis (AAV)	16 yy F	Demir et al., 2021 [37]
Resolution of fever	Surgical	Present	Elevated	Elevated	Yes	Mass in the rectovesical pouch	9 yy M	Chakrabarti et al., 2019 [38]
Resolution of mass and normalization of APR	Prednisolone, MMF then Rituximab	Present	Elevated	Elevated	No	Swelling in the upper arm	14 yy F	Özdel et al., 2020 [39]
	Steroids	Present	Elevated		No	Pancreatitis Sclerosing cholangitis IBD and lacrimal gland involvement	7 yy F	Akkelle et al., 2020 [40]
	Surgical	Present	Normal		Yes	Pneumonia Posterior pulmonary consolidated mass lesion	7 yy M	Szczawinska-Poplonyk et al., 2016 [41]
Resolution of mass after 1 year follow up	Steroids	Present	Elevated	Normal	No	Bilateral submandibular swelling	16 yy M	Ferreira da Silva et al., 2017 [42]
NR	NR	Present	NR	Elevated	No	Orbital disease	3 yy M	Raab et al., 2018 [43]
	Prednisolone	Present	Elevated		No	Tracheal stenosis	17 yy F	Gabrovska et al., 2021 [44]
Remission	Short course dexamethasone	Present	Normal	Elevated	No	Left parotid swelling	6 yy M	Timeus et al., 2021 [45]
Remission	Surgical	Present	Elevated	NR	No	Orbital disease	8 yy F	Hoshiyama et al., 2022 [46]
Remission	Steroids, AZA, TMP-SMX prophylaxis then MMF	Present	Elevated	Elevated	No	Orbital disease	15 mm M	Tong et al., 2021 [47]
NR	NR	Present	Elevated	Normal	NR	Unilateral orbital disease Headache, proptosis	14 yy F	Kaya Akca et al., 2021 [17]
NR	NR	Present	Elevated	Normal	NR	Unilateral orbital disease Lacrimal gland swelling	13 yy M	
NR	NR	Present	Elevated	Normal	NR	Unilateral orbital disease Proptosis	10 yy M	
NR	NR	Present	NR	Normal	Yes	Unilateral orbital disease Eyelid tenderness, small pulmonary nodule	13 yy F	
NR	NR	Present	Elevated	Elevated	NR	Unilateral orbital disease Proptosis, 5th cranial nerve affection	9 yy F	
NR	NR	Present	Elevated	Elevated	NR	Abdominal pain, mesenteric lymphadenopathy	4 yy M	
NR	NR	Present	Elevated	Normal	Yes	Abdominal pain Salivary gland swelling, Ulcerative colitis, lymphadenopathy	15 yy M	
NR	Steroids	Present	Elevated	NR	NR	Autoimmune pancreatitis	13 yy M	Miglani et al., 2010 [48]
Relapsed after tapering, required low dose maintenance Steroids and AZA	Steroids, AZA	Present	Elevated	NR	NR	Cholangitis	3 yy F	Ibrahim et al., 2011 [49]
No relapse after tapering and stoppage of Steroids and MMF	Steroids, MMF	Present	Elevated	NR	NR	Autoimmune pancreatitis, fibrosing mediastinitis, renal and hepatic affection	13 yy F	Mannion & Cron, 2011 [50]
Steroids tapered and stopped in 3 months	Steroids	Present	NR	NR	NR	Riedel’s thyroiditis	17 yy M	Zakeri & Kashi, 2011 [51]
NR	Steroids	Present	NR	NR	NR	Sialadenitis	11 yy M	Melo et al., 2012 [52]
Refractory to Steroids and rituximab but responded to adalimumab	Adalimumab	Present	Elevated	NR	NR	Colitis Autoimmune pancreatitis	16 yy F	Naghibi et al., 2013 [53]
4 weeks	Steroids	Present	Elevated	NR	NR	Pulmonary disease	15 yy M	Pifferi et al., 2013 [54]
Relapse	Steroids, rituximab	Present	Normal	NR	NR	Orbital disease Nephrotic syndrome	12 yy F	Sane et al., 2013 [55]
Refractory to MMF, but responded to Rituximab	Steroids, rituximab	Present	Elevated	NR	NR	Lymphadenitis Scleritis	17 yy M	Caso et al., 2014 [56]
Relapsed on AZA, needed maintenance Steroids	Steroids, AZA, colchicine	Present	Elevated	NR	NR	Mesenteritis pancreatitis	7 yy F	Hasosah et al., 2014 [57]
Good response	Steroids, AZA	Present	Elevated	NR	NR	Orbital disease	7 yy M	Jariwala et al., 2014 [58]
Initial improvement	Steroids	Present	Elevated	NR	NR	Orbital disease	14 yy M	Mittal et al., 2014 [59]
NR	Steroids	Present	Normal	NR	NR	Orbital disease (dacryoadenitis)	13 yy F	Notz et al., 2014 [60]
NR	Steroids, Rituximab	Present	Elevated	NR	NR	Orbital disease Sinonasal disease	15 yy F	Prabhu et al., 2014 [61]
NR	Steroids	Present	Elevated	NR	NR	Orbital disease	15 yy F	
NR	Steroids, MTX	Present	Elevated	NR	NR	Orbital disease	14 yy F	Batu et al., 2015 [13]
NR	Steroids, cyclophosphamide	Present	Elevated	NR	NR	Orbital disease-	9 yy F	
Responded to rituximab	Steroids, rituximab	Present	Normal	NR	NR	Orbital disease Renal disease	7 yy F	Gillispie et al., 2015 [62]
Coagulopathy improved after Steroids	Steroids	Present	Elevated	NR	NR	Hepatic mass Coagulopathy	10 yy M	Nada et al., 2015 [63]
Regression	None	Present	Elevated	Normal	NR	Lymphadenopathy	14 yy M	Meli et al., 2023 [64]
Regression	None	Present	Elevated	Elevated	Yes	Abdominal lymphadenopathy	16 yy M	
Regression	Pancreaticoduodenectomy	Present	Elevated	Normal	Yes	Duodenal stenosis and ulceration	12 yy F	Kato et al., 2023 [65]
NR	NR	Present	Elevated	NR	NR	Cervical lymphadenopathy	13 yy F	Ewing et al., 2016 [66]
Relapse with tapering steroids, iatrogenic cushing with high doses steroids, regression with MMF	Steroids, MMF	Present	Elevated	NR	No	Orbital disease (dacryoadenitis)	9 yy F	Rojas-Ramirez et al., 2016 [67]
Regression	Surgical resection of the left upper lobe, Steroids, rituximab	Present	Elevated	Normal	Yes	Lung upper lobe mass, recurrent respiratory infections, abdominal lymphadenopathy, pleural and pericardial effusion	3 yy M	Marissen et al., 2021 [68]
-	-	Present	Normal (but elevated total IgG)	Elevated	Yes	Cough, dyspnea, nasopharyngeal mass, hepatitis	15 yy F	Woo et al., 2021 [69]
Regression	Steroids	Present	Elevated	Elevated	Yes	Lumbar pain, spondilodiscitis	15 yy M	Zeybeck et al., 2021 [70]
Regression	Steroids + Cellcept + adalimumab	Present	NR	NR	Yes	Coronary artery involvement, orbital disease	15 yy F	Mohammadzadeh et al., 2023 [71]
Regression	Steroids, MMF	Present	Elevated	Yes	Yes	Acute tubulointerstitial nephritis	16 yy M	Pac et al., 2023 [72]
Regression	Steroids, AZA / MMF	Present	Elevated	NR	NR	Orbital disease	Mean 7 yy 2 F	Singla et al., 2023 [22]
Regression	Steroids, AZA	Present	Elevated	NR	NR	Orbital disease, sialadenitis	11 yy M	Rodrigues et al., 2023 [73]
NR	NR	Present	Elevated	NR	NR	Orbital disease	13 yy M	Hsueh et al., 2023 [74]
Recurrence	Total enteral nutrition	Present	Elevated	Normal	NR	Duodenal ulcer	14 yy M	Ma et al., 2022 [75]
NR	Surgical, steroids	Present	NR	NR	Yes	Orbital disease	7 yy M	Farha et al., 2023 [76]
Rapid clinical improvement	Surgical, steroids	Present	NR	NR	NR	Gastric desmoid fibromatosis, urethral lesion	13 yy M	Niksic et al., 2023 [77]
Regression	Steroids, MMF	Present	Elevated	Elevated	Yes	Renal disease	7 yy M	Tsygin et al., 2022 [78]
Regression	Steroids, MMF	Present	Elevated	Normal	No	Orbital disease, central and peripheral nervous system involvement	14 yy F	Qing et al., 2022 [79]
Regression	Steroids, MMF, surgical	Present	Normal	Normal	No	Orbital disease	12 yy F	Kasap-Demir, 2022 [80]
Relapse with tapering Steroids, regression with rituximab	Steroids, rituximab	Present	Elevated	Normal	No	Orbital disease	8 yy F	Qi et al., 2022 [81]
NR	Steroids	Present	NR	NR	No	Intracranial hypertrophic pachymeningitis, sclerosing sialadenitis and orbital disease	8 yy M	De Jesus et al., 2021 [82]
Regression	Deflazacort	Present	Elevated	Elevated	Yes	Coronary artery aneurysm	13 yy M	Vasudevan et al., 2021 [83]
Regression	Steroids, AZA, mesalamine, UDCA	Present	Elevated	Normal	Yes	Sclerosing cholangitis and ulcerative colitis	3 yy M	Hsu et al., 2020 [84]
Regression with rituximab	Steroids, MTX, MMF, rituximab	Present	Normal	Normal	NR	Retroperitoneal fibrosis	11 yy F	Raja et al., 2020 [85]
No response	Steroids	Present	Elevated	NR	NR	Kidney disease	2 yy	La Porta et al., 2020 [86]
NR	Steroids	Present	NR	NR	NR	Orbital disease	14 mm M	Tanzifi et al., 2020 [87]
Rapid clinical improvement	Steroids, rituximab, MMF	Present	Elevated	NR	Yes	Orbital disease, sialadenitis	10 yy F	Cinar et al., 2019 [88]
Regression	Steroids, AZA	Present	Elevated	Elevated	Yes	Hepatic mass	10 yy M	Kumar et al., 2019 [89]
Regression	Steroids, AZA	Present	Elevated	NR	NR	Intragastric mass	12 yy F	
Regression	Steroids, AZA	Present	Elevated	Elevated	NR	Erythematous swellings over dorsum of the left hand, forearm and chest	14 yy M	
Regression	Steroids, AZA	Present	Elevated	Elevated	Yes	Retroperitoneal fibrosis	18 yy F	
Regression	Steroids	Present	Elevated	Elevated	Yes	Tubulointerstitial nephritis	7 yy F	
Regression	Steroids	Present	Elevated	NR	NR	Orbital disease	14 yy M	
Regression	Surgery	Present	NR	NR	No	Kidney mass	11 yy M	Johnson et al., 2018 [90]
NR	NR	Present	NR	Elevated	No	Orbital disease	9 yy F	Deepak et al., 2018 [91]
Regression	Rituximab, Sirolimus, ruxolitinib, surgery	Present	NR	NR	NR	Orbit, hip muscle, peripancreatic tissue involvement, polylymphadenopathy, pulmonary, renal and hepatic foci	13 yy 1F, 4 M	Kozlova et al., 2018 [92]
Regression	Steroids	Present	Elevated	Elevated	NR	Multiple lymphadenopathies	9 yy M	Chen et al., 2018 [93]
Regression	Steroids, surgery	Present	Elevated	NR	No	Orbital disease	12 yy M	Parvaneh et al., 2018 [94]
Rapid clinical improvement	Steroids, Rituximab	Present	NR	NR	No	Orbital disease	9 yy F	Eng et al., 2017 [95]
Regression	Steroids, MMF	Present	Elevated	Elevated	No	Orbital disease	10 yy F	Ozdemir et al., 2017 [96]
Regression	Steroids, MMF	Present	Normal	Elevated	Yes	Orbital disease	16 yy F	Diaz et al., 2017 [97]
Regression with MMF	Steroids, MMF	Present	Elevated	Normal	NR	Orbital disease, cholangitis, cholecystitis and nephropathy	12 yy F	Okamoto et al., 2017 [98]
Rapid clinical and radiological improvement	Steroids, AZA, surgery	Present	Elevated	Normal	No	Massive Pleural Effusion, Mediastinal Mass, and Mesenteric Lymphadenopathy	16 yy M	Goag et al., 2015 [99]
Regression	Surgery, steroids	Present	Elevated	Elevated	Yes	Appendicitis	17 yy M	Cabrales-Escobar et al., 2020 [100]
Regression	Surgery	Present	Elevated	Elevated	No	Soft tissue mass	16 yy M	Creze
								et al., 2019 [101]
Rapid clinical and radiological improvement	Steroids	Absent	Elevated	Normal	No	Orbital disease, left maxillary sinus, temporal, and masseter muscles, zygomatic and frontal bones, dural thickening.	12 yy F	Our first case
Recurrence of disease with tapering of the corticosteroids	Steroids	NR	Elevated	Normal	No	Orbital disease (dacryoadenitis)	14 yy M	Our second case

Abbreviations: yy: years old, mm: months old, F: female, M: male, NR: not reported, MTX: methotrexate, AZA: azathioprine, UDCA: Ursodeoxycholic acid, MMF: mycophenolate mofetil, TMP-SMX: trimethoprim/sulfamethoxazole.

## Data Availability

No new data were created or analyzed in this study. Data sharing is not applicable to this article.

## Abbreviations

ACR: American College of Rheumatology, AAV: ANCA Associated vasculitis, AIHA: Autoimmune hemolytic anemia, ANA positive antinuclear antibodies, AZA: azathioprine, CDC: Comprehensive Diagnostic Criteria, CRP: C-reactive protein, CSF: Cerebrospinal fluid, CT: computed tomography, ESR: Erythrocyte Sedimentation Rate, EULAR: European League Against Rheumatism, F: female, GCs: Glucocorticoids, IBD: Inflammatory bowel disease, IgG4-HP: Immunoglobulin G4 Hypertrophic pachymeningitis, IgG4-RD: Immunoglobulin G4-related disease, IgG4-ROD: Immunoglobulin G4-related orbital disease, IMRAD: Introduction, Methods, Results, and Discussion, IRAK-4: interleukin-1 receptor-associated kinase 4, LFTs: liver function tests, M: male, mm: months old, MMF: mycophenolate mofetil, MRI: magnetic resonance imaging, MTX: methotrexate, TMP-SMX: trimethoprim/sulfamethoxazole, RCD: revised comprehensive diagnostic, RF: Rheumatoid Factor, UDCA: Ursodeoxycholic acid.
